# Differential expression of Mediator complex subunit MED15 in testicular germ cell tumors

**DOI:** 10.1186/s13000-015-0398-6

**Published:** 2015-09-17

**Authors:** Niklas Klümper, Isabella Syring, Anne Offermann, David Adler, Wenzel Vogel, Stefan C. Müller, Jörg Ellinger, Arne Strauß, Heinz Joachim Radzun, Philipp Ströbel, Johannes Brägelmann, Sven Perner, Felix Bremmer

**Affiliations:** 1 Section for Prostate Cancer Research, Institute of Pathology, Center for Integrated Oncology Cologne/Bonn, University Hospital of Bonn, Bonn, Germany; 2 Department of Urology and Pediatric Urology, University Hospital of Bonn, Bonn, Germany; 3 Department of Urology, University Medical Center, University of Göttingen, Göttingen, Germany; 4 Institute of Pathology, University Hospital of Göttingen, Robert Koch Str. 40, 37075 Göttingen, Germany; 5 Department of Hematology/Oncology, University Hospital of Bonn, Bonn, Germany

**Keywords:** Seminoma, Embryonic Carcinoma, Testicular Germ Cell Tumor, MED15 Expression, Mediator Complex

## Abstract

**Background:**

Testicular germ cell tumors (TGCT) are the most common cancer entities in young men with increasing incidence observed in the last decades. For therapeutic management it is important, that TGCT are divided into several histological subtypes. MED15 is part of the multiprotein Mediator complex which presents an integrative hub for transcriptional regulation and is known to be deregulated in several malignancies, such as prostate cancer and bladder cancer role, whereas the role of the Mediator complex in TGCT has not been investigated so far. Aim of the study was to investigate the implication of MED15 in TGCT development and its stratification into histological subtypes.

**Methods:**

Immunohistochemical staining (IHC) against Mediator complex subunit MED15 was conducted on a TGCT cohort containing tumor-free testis (*n* = 35), intratubular germ cell neoplasia unclassified (IGCNU, *n* = 14), seminomas (SEM, *n* = 107) and non-seminomatous germ cell tumors (NSGCT, *n* = 42), further subdivided into embryonic carcinomas (EC, *n* = 30), yolk sac tumors (YST, *n* = 5), chorionic carcinomas (CC, *n* = 5) and teratomas (TER, *n* = 2). Quantification of MED15 protein expression was performed through IHC followed by semi-quantitative image analysis using the *Definiens* software.

**Results:**

In tumor-free seminiferous tubules, MED15 protein expression was absent or only low expressed in spermatogonia. Interestingly, the precursor lesions IGCNU exhibited heterogeneous but partly very strong MED15 expression. SEM weakly express the Mediator complex subunit MED15, whereas NSGCT and especially EC show significantly enhanced expression compared to tumor-free testis.

**Conclusions:**

In conclusion, MED15 is differentially expressed in tumor-free testis and TGCT. While MED15 is absent or low in tumor-free testis and SEM, NSGCT highly express MED15, hinting at the diagnostic potential of this marker to distinguish between SEM and NSGCT. Further, the precursor lesion IGCNU showed increased nuclear MED15 expression in the preinvasive precursor cells, which may provide diagnostic value to distinguish between benign and pre-malignant testicular specimen, and may indicate a role for MED15 in carcinogenesis in TGCT.

## Background

In young men at the age of 15 to 40 years, testicular germ cell tumors (TGCT) are the most frequent malignant tumors [1]. Interestingly, an increasing incidence in TGCT has been observed over the last 40 years [2]. TGCT are histologically and clinically grouped into seminomas (SEM) and non-seminomatous germ cell tumors (NSGCT), which are further subdivided into embryonic carcinomas (EC), yolk sac tumors (YST), chorionic carcinomas (CC), and teratomas (TER) [3]. Generally, NSGCT have a more aggressive and undifferentiated phenotype than SEM and tend to be metastatic [4]. Therefore, the histological distinction between these tumor subentities plays an important role for the therapeutic management and new biomarkers are needed for higher sensitivity in diagnostics.

MED15 is part of the multiprotein Mediator complex (MED) and serves as a hub for important signaling pathways, transcriptional co-activators and co-repressors forming a bridge between the RNA polymerase II (Pol II) and transcriptional factors [5, 6]. The Mediator complex has frequently been described to be differentially expressed or mutated in diverse tumor entities [7]. Interestingly, MED15 belongs to the tail module of the Mediator complex, which is known to receive and integrate information from diverse signaling pathways such as the transforming growth factor-β (TGF-β) and sterol regulatory element-binding protein (SREBP) pathways [8–10]. While some cancer entities were shown to be strongly associated with MED deregulation, knowledge about the Mediator complex expression profile in TGCT has been lacking so far.

We therefore evaluated the MED15 expression in tumor-free testis and TGCT subentities by immunohistochemical staining (IHC) on a large tissue microarray (TMA) cohort in order to evaluate a possible diagnostic and therapeutic value for MED15 in TGCT.

## Methods

### Tissue samples of primary TGCT

In this study tissue microarrays (TMA) containing specimens of testes were used to examine the MED15 protein expression by immunohistochemical analysis. The TGCT cohorts, kindly provided by the Institute for Pathology Göttingen and the Urology Department of the University Hospital Bonn, include the following tissue samples: 35 tumor-free testes, 14 intratubular germ cell neoplasia unclassified (IGCNU), 107 seminomas (SEM) and 42 non-seminomatous germ cell tumors (NSGCT), further subdivided into embryonic carcinomas (EC, *n* = 30), yolk sac tumors (YST, *n* = 5), chorionic carcinomas (CC, *n* = 5) and teratomas (TER, *n* = 2). Ethical approval for using human material in the present study was obtained from the ethics committee of the University Medical Centre Göttingen and Bonn.

The TMA was constructed as described previously [11]. Briefly, formalin-fixed paraffin-embedded tissues were cut into 4 μm thick sections and were mounted on slides. After staining with haematoxylin and eosin (H&E), areas of normal tissue and primary tumor were determined and circled by a pathologist. Representative cores of the circled regions, measuring 0.6 mm in diameter from each formalin-fixed paraffin-embedded (FFPE) benign tissue and primary tumor, were assembled into tissue microarray blocks (recipient blocks) using a semiautomatic tissue arrayer (Beecher Instruments, Sun Prairie, WI, USA). H&E TMA sections were assessed again to confirm the histology of the selected regions.

### Immunohistochemical analysis (IHC)

Immunohistochemical staining was conducted using the Ventana Benchmark automated staining system (Ventana Medical System, Tuscon, AZ, USA). In brief, slides were incubated at room temperature with the primary antibody according to the manufacturer (dilutions, clones, manufacturer): anti-MED15 rabbit polyclonal (1:100, 11566-1-AP, Proteintech, Chicago, IL, USA). Antibody dilution was conducted using a Ventana diluent. Signal detection was done using the ultraView Universal DAB detection kit (Ventana Medical System, Tuscon, AZ, USA). Finally, slides were counterstained with haematoxylin and bluing reagent, dehydrated, and mounted. IHC stainings were validated independently by two pathologists (F.B., S.P). Only cases with at least one assessable core were included in this analysis. Tumor samples with a lack of tissue or absence of carcinoma were excluded.

### Quantification of protein expression

Quantification of MED15 protein expression was performed using the semi-quantitative image analysis program *Definiens* (Definiens Inc., Munich, Germany). Hereby, the pathologist chose manually the tumor area within the testicular specimens. Afterwards, the program analyzed the selected regions of interest with respect to overall protein expression using the average staining intensity (mean brown chromogen intensity) and the number of positively stained cells determined through an intensity threshold in relation to all analyzed cells in a sample (positive index). The statistical evaluation of the expression intensity was performed using the two-sided Student’s *t*-test in *SPSS Statistics 22* (SPSS Inc., Chicago, IL, USA).

## Results

### MED15 expression in tumor-free testes, precursor lesions, seminomas and non-seminomatous germ cell tumors

**Fig. 1:**
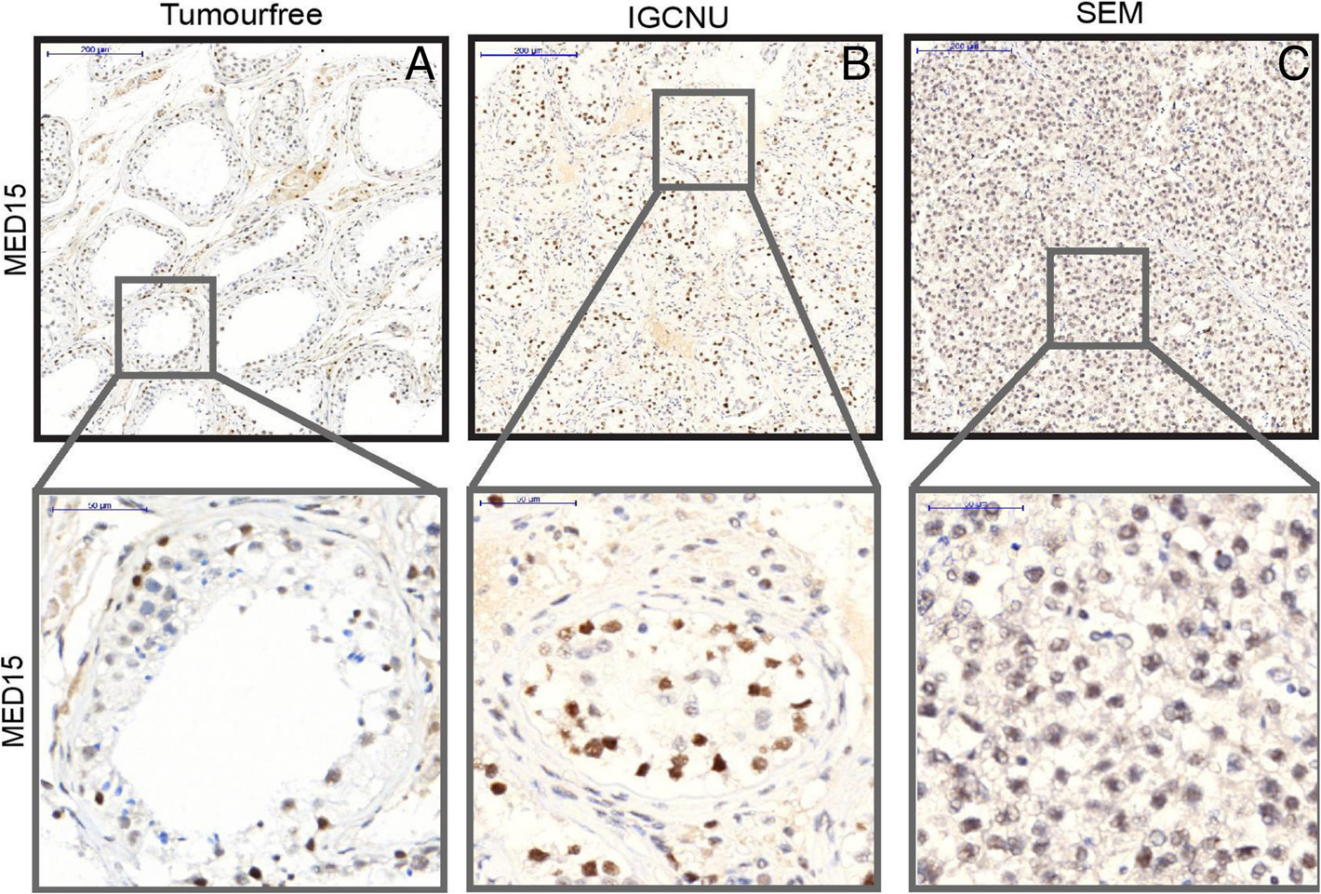
Representative IHC images for MED15 expression from tissue of tumor-free testis (**a**), intratubular germ cell neoplasia unclassified (IGCNU) (**b**) and seminoma (SEM) (**c**). 10× (upper panel) and 40× (lower panel) objective magnification

In tumor-free testes the MED15 protein expression was absent or low. Interestingly, pre-eminently the pluripotent spermatogonia exhibit moderate MED15 expression in the tumor-free testis (Fig. 1a). Intratubular germ cell neoplasia unclassified (IGCNU) showed increased nuclear MED15 expression in the preinvasive precursor cells and an increased positive index (Fig. 1b).

**Fig. 2:**
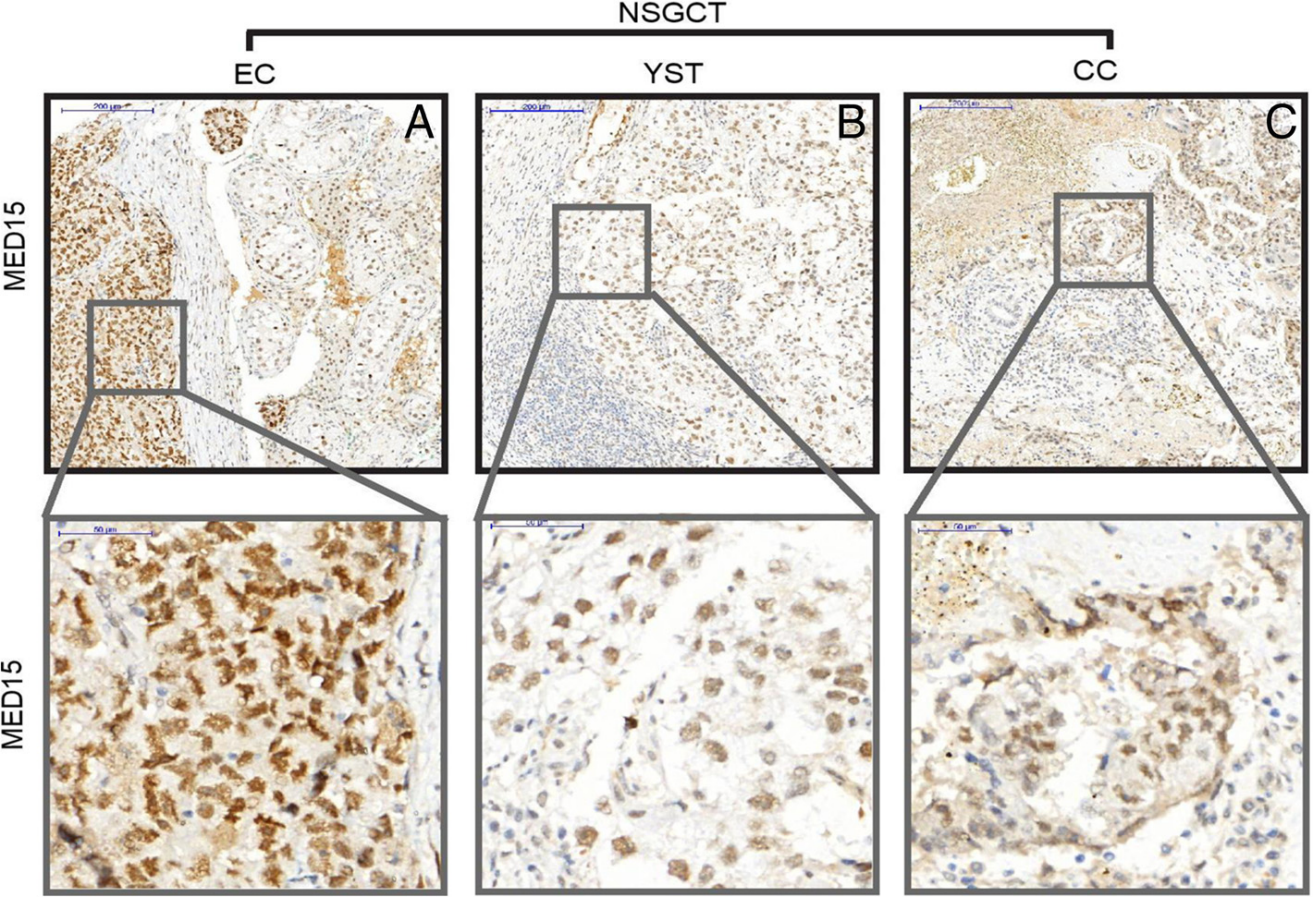
Representative IHC images for MED15 expression from tissue of the different non-seminomatous germ cell tumors (NSGCT) embryonic carcinoma (EC) (**a**), yolk sac tumor (YST) (**b**) and chorionic carcinoma (CC) (**c**). 10× (upper panel) and 40× (lower panel) objective magnification

**Fig. 3:**
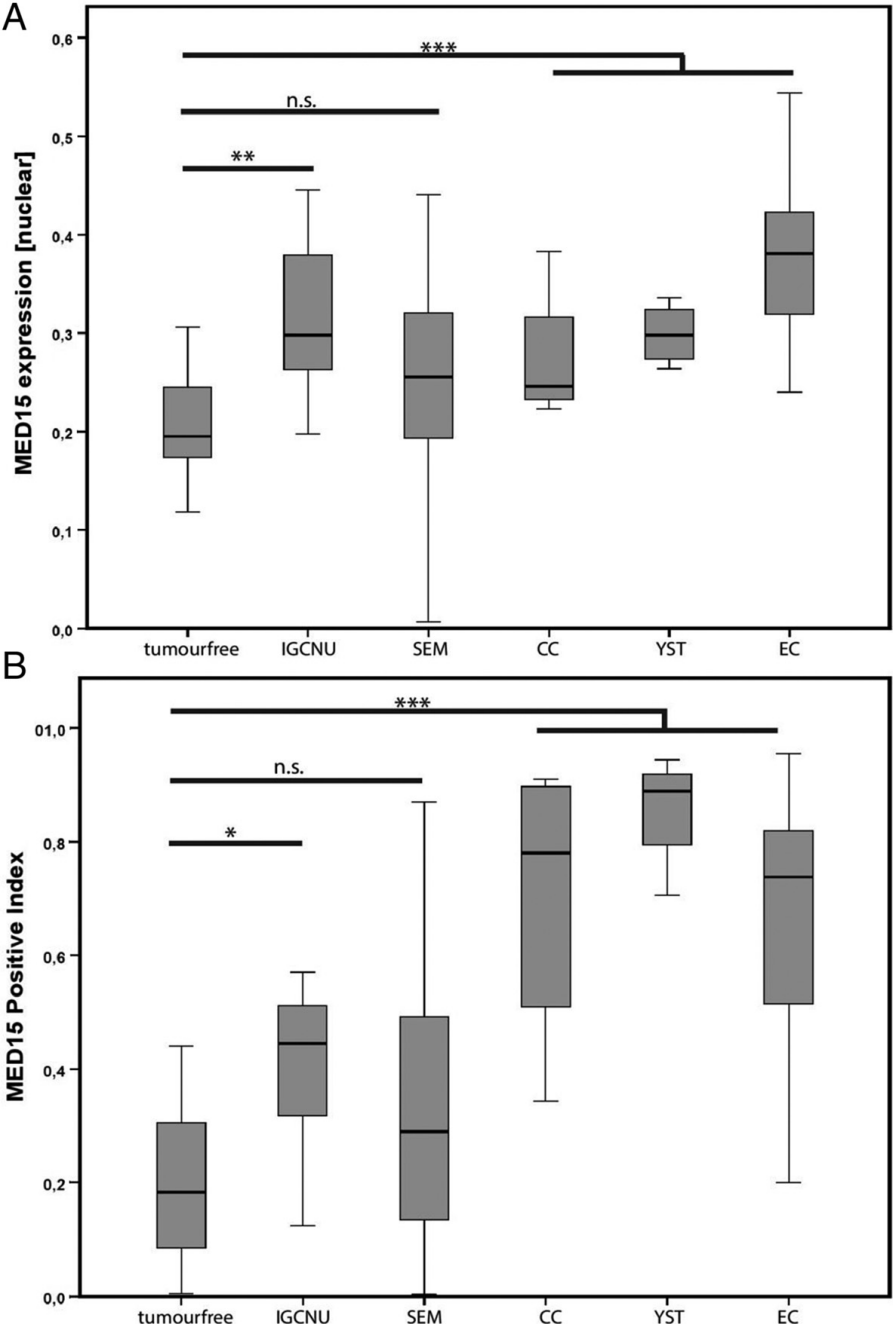
**a** Nuclear MED15 protein expression profile of the total testicular germ cell tumor (TCGT) cohort. **b** Positive index of the MED15 immunhistochemical staining on the TGCT cohort. (n.s. = not significant, * = *p* < 0.05, ** = *p* < 0.01, *** = *p* < 0.001)

In SEM, MED15 expression was predominantly low or completely absent (Fig. 1c). In contrast, non-seminomatous germ cell tumors (NSGCT) exhibited a significantly higher MED15 expression and positivity index as compared to tumor-free testes and SEM. Especially, the staining pattern in EC showed strong homogeneous expression of MED15 in the nuclei and cytoplasm (Fig. 2a). Likewise, samples of NSGCT, YST (Fig. 2b) and CC (Fig. 2c) exhibited elevated nuclear and cytoplasmic MED15 expression as compared to tumor-free testes and SEM. TER were found with high diversity in MED15 expression in line with their histologic heterogeneity (data not shown). In conclusion, the NSGCT significantly overexpress MED15 as compared to tumor-free testes and SEM (*p* < 0.001). Further, IGCNU as a pre-malignant precursor lesion of the testes showed enhanced MED15 protein expression as compared to benign tissue (*p* < 0.01) (Fig. 3a). Interestingly, NSGCT showed an elevated MED15 positive index indicating a homogeneous expression in the tumor, whereas tumor-free testes and SEM showed significantly lower positive indexes (*p* < 0.001) (Fig. 3b).

## Discussion

In this study, we found MED15 nuclear and cytoplasmic expression to be significantly higher in NSGCT as compared to tumor-free testes and SEM. Further, the precursor lesion intratubular germ cell neoplasia unclassified (IGCNU) exhibited increased nuclear MED15 expression in the preinvasive precursor cells as compared to benign tissue which may be a hint for MED15 overexpression as a clonal event in TGCT development. Interestingly, MED15 was found to be homogeneously expressed in NSGCT (positive index >0.8) pointing at a possible role for MED15 in selection of precursor tumor cells during carcinogenesis. MED15 is part of the Mediator complex, which is an evolutionarily conserved multi-protein assembly. It plays a pivotal role in transcriptional regulation forming a bridge between transcriptional factors and the RNA polymerase II (Pol II) [5]. The complex can be subdivided into four distinct submodules termed the head, middle, tail and kinase. While the head, middle and tail module form a stable core complex, the kinase module associates reversibly with the core complex thereby influencing the activity of the whole complex [6]. Generally, the head and middle modules interact directly with Pol II, the main part of the core associated basal transcription machinery, whereas the tail and kinase modules serve as major loci for signal transduction and diverse signaling pathways [5].

Genomic alteration as well as altered expression of distinct Mediator complex subunits (MED) can cause dysfunctions and dysregulations of cell signaling and may contribute to malignant properties through directly mediating target gene transcription [7]. Diverse MED subunits have been associated with tumor development, progression and the emergence of chemoresistance, which remains one of the key problems in oncologic therapy. Even though modern therapeutical options for TGCT such as radical orchiectomy and chemotherapy achieve high cure rates, a small group of patients nevertheless experiences late relapse, metastatic spread or chemoresistance which leads to poor prognosis and underlines the importance for unraveling the responsible mechanisms [12].

MED15 is part of the tail module and is known to serve as a hub for signaling pathways including transforming growth factor-β (TGF-β) signaling as well as the signaling of sterol regulatory element-binding proteins (SREBP) [8, 9]. The TGF-β signaling pathway is a double-edged sword, with either tumor suppressive or oncogenic features depending on the stage of disease [10]. For example, in castration-resistant prostate cancer, MED15 is frequently overexpressed and correlates with pSmad3 expression, a downstream target indicating activation of the TGF-β signaling cascade [13]. MED15 was further described to retain TAZ (transcriptional co-activator with PDZ-binding motif) in the nucleus, which engages in shuttling Smad complexes and dominantly regulates Smad nuclear accumulation, thereby positively regulating TGF-β target gene expression. In human embryonic stem cells MED15 is required for stem cell maintenance and pluripotency through direct interaction with TAZ leading to Smad nuclear accumulation and target gene transcription, whereas loss of TAZ leads to inhibition of TGF-β signaling and differentiation. This is in line with the results presented in the current study, which shows a significantly enhanced MED15 expression in pluripotent EC [14]. Interestingly, treatment of the seminoma cell line TCam-2 with recombinant TGF-β1 induces differentiation into a cell type resembling mixed non-seminoma [15] suggesting MED15 as an integrative hub for this differentiation process.

In addition, activated TGF-β signaling leads to induction of epithelial-to-mesenchymal transition, which is known to be implicated in tumor progression, metastatic spread and increased chemoresistance [16, 17, 10]. Thus, positive regulation of TGF-β signaling might indicate a role of MED15 in the emerging of an aggressive phenotype. Therefore, enhanced MED15 protein expression found in TCGT could be a hint for non-responsive and invasive tumors and requires further investigation. Previously, the SREBP target gene fatty acid synthase (FASN), which is a key enzyme responsible for the endogenous synthesis of fatty acids and described to be upregulated in different tumors, was detected to be differentially expressed in TCGT. Especially, EC frequently overexpress FASN, whereas SEM and tumor-free testes express FASN rarely [18]. Considering the strong overexpression of MED15 in TGCT and EC as presented here, the involvement of the MED15-SREBP-FASN axis in tumor formation and differentiation should be investigated in detail with the aim to potentially develop diagnostic biomarker and identify novel therapeutic targets.

## Conclusions

In conclusion, the differential protein expression of Mediator complex subunit MED15 in TGCT may provide valuable as a diagnostic marker and may assist the selection of therapeutic intervention. MED15 is expressed significantly higher in precursor lesion IGCNU as compared to tumor-free testes and may thus prove useful as a diagnostic feature to distinguish between benign and pre-malignant tissue. Furthermore, MED15 may play a role in the differentiation process between seminomas and non-seminomas.

## Abbreviations

TGCT: Testicular germ cell tumors
SEM: Testicular seminomas
NSGCT: Non-seminomatous germ cell tumors
EC: Embryonic carcinomas
YST: Yolk sac tumors
CC: Chorionic carcinomas
TER: Teratomas
MED15: Part of the multiprotein Mediator complex
TGF-β: transforming growth factor-β
SREBP: Sterol regulatory element-binding protein
TAZ: Transcriptional co-activator with PDZ-binding motif
FASN: Fatty acid synthase
